# Deep Learning with Multimodal Integration for Predicting Recurrence in Patients with Non-Small Cell Lung Cancer

**DOI:** 10.3390/s22176594

**Published:** 2022-08-31

**Authors:** Gihyeon Kim, Sehwa Moon, Jang-Hwan Choi

**Affiliations:** 1 Computational Medicine, Graduate Program in System Health Science and Engineering, Ewha Womans University, Seoul 03760, Korea; 2 Division of Mechanical and Biomedical Engineering, Graduate Program in System Health Science and Engineering, Ewha Womans University, Seoul 03760, Korea; 3 Department of Artificial Intelligence, Ewha Womans University, Seoul 03760, Korea

**Keywords:** clinical feature, handcrafted radiomics, deep learning-based radiomics, non-small cell lung cancer, cancer recurrence

## Abstract

Due to high recurrence rates in patients with non-small cell lung cancer (NSCLC), medical professionals need extremely accurate diagnostic methods to prevent bleak prognoses. However, even the most commonly used diagnostic method, the TNM staging system, which describes the tumor-size, nodal-involvement, and presence of metastasis, is often inaccurate in predicting NSCLC recurrence. These limitations make it difficult for clinicians to tailor treatments to individual patients. Here, we propose a novel approach, which applies deep learning to an ensemble-based method that exploits patient-derived, multi-modal data. This will aid clinicians in successfully identifying patients at high risk of recurrence and improve treatment planning.

## 1. Introduction

Non-small cell lung cancer (NSCLC), which accounts for 85% of lung cancer cases [1], is one of the most common and fatal cancer types worldwide [2]. While the most common treatment strategy for NSCLC patients is surgery, a high probability of tumor recurrence following surgical resection typically results in a bleak prognosis [3]. In fact, early-stage NSCLC patients (Stage I, II, IIIA) who underwent resection surgery have recurrence rates of 40% (stage I), 66% (stage II) and 75% (stage IIIA) [4,5,6]. Therefore, the ability to accurately predict NSCLC relapse is extremely critical considering that, as cancer progresses, the five-year survival rates decrease sharply, from 40% of stage I patients to only 1% of stage IV patients [7,8]. Thus, the ability to identify patients with a high risk for recurrence following surgical resection is critically important because it allows clinicians to determine which patients may benefit from adjuvant therapies [9], and thus create more effective personalized treatments.

However, for patients with NSCLC, crafting treatment strategies that include adjuvant therapies is difficult [10,11]. Of the several common factors known to be associated with NSCLC relapse in early NSCLC, such as tumor size (T-stage), nodule involvement (N-stage) and smoking history [12,13,14], the tumor node (TN) staging system is traditionally used as a postoperative prognostic factor [15,16]. The TN staging system provides medical professionals with a framework for understanding the prognosis, treatment options, and value of new interventions allowing for the best possible care for lung cancer patients [17]. However, because tumor size is determined qualitatively, accurate TN staging can be limited due to human error [18]. The TN staging system is further limited in predicting clinical outcomes due to the lack of a clear rank order by stage [19]. Thus, the current staging system for NSCLC is insufficient for predicting treatment outcomes, illustrating the critical need for a novel predictive model. Moreover, an alternative model must possess a high predictive power to identify patients at high risk of relapse and help guide clinical decisions for optimal treatment.

One of the key resources that shape a patient’s clinical trajectory is the use of non-invasive medical imaging, and imaging is widely accepted as a standard procedure for treatment in routine clinical practice [20,21]. In particular, computed tomography (CT) texture analysis can visualize intra-tumor heterogeneity, which may result in disparate patient outcomes [22]. This imaging technology can aid clinicians in the planning and execution of treatment strategies. However, complex pathological characteristics can often be overlooked by normal imaging techniques. To discover tumor characteristics that may not be visualized by the naked eye, radiomics, a visual imaging technique that has traditionally made use of handcrafted radiomic features (HCR), has been widely utilized as an imaging biomarker to predict clinical outcomes and therapy responses [21,23]. Under the biological assumption that distinct phenotypic morphologies can be visualized using high specialized imaging techniques, high throughput of quantitative descriptors including intensity distribution, spatial relationships, texture heterogeneity patterns, and volumetric quantification are being used as input data for statistical or standard machine learning models [20,24,25]. However, traditional radiomic methods have recently come under question due to possible perceived human bias during analysis [26]. Therefore, deep learning-based radiomics (DLR) with convolution layers has been developed as a method to extract latent pathological features. Data passed through the convolutional neural network (CNN) is non-linearly mapped throughout the transformation linking the input and output spaces of networks, allowing potential features projected into the synthetic feature space at each layer to be investigated in relation to the outcome label [20]. In other words, using a deep learning paradigm enables automatic learning of the relevant radiographic features without prior definition by researchers, which connotes greater learning capabilities, enhanced generality, and accuracy.

In this paper, we propose an ensemble-based prediction model for NSCLC recurrence after surgical resection. First, we constructed three neural network models, each trained with (1) clinical data including TN stage, (2) HCR features, and (3) DLR features, and optimized for each data-based prediction. Then, the integrated predictive outputs of the three models went through a different machine learning-based ensemble analyzer for making the final decision. This may allow for compensation of shortcomings of the three prior models. Several previous NSCLC studies conducted, including a systematic review, have identified CT-based image features that are related to recurrences, such as a sequentially enlarging mass-like lesion, opacity enlargement after 12 months, filling-in of air bronchograms, bulging margins, the disappearance of the linear margin, development of ipsilateral pleural effusion, and lymph node enlargement [27,28]. While we expect HCR and DLR networks to perform predictive functions that take into account these external NSCLC tumor characteristics, the key factors that exist outside of the medical image, such as nodal involvement [29], were not analyzed. Therefore, the proposed ensemble model of concatenating inferences from pathological information and medical image features has high potential to produce refined and precise results.

The main contributions of this paper are as follows:

- Neural network-based recurrence prediction models exploiting (1) clinical information, (2) radiomics, and (3) deep learning-based features, respectively, were developed.
- The performance of ensemble models using various combinations of the three patient-derived multi-modal features was evaluated.
- The ensemble model using all three features showed the best performance.
- A first recurrence prediction model for early NSCLC through the integration of the aforementioned three features was proposed.

## 2. Materials and Methods

### 2.1. Patient Data Acquisition

Our patient data was provided by two separate institutions. One is the Veterans Health Service (VHS) Medical Center, a hospital that treats ordinary citizens to national merit. The other institution we obtained data from was the Cancer Imaging Archive (TCIA) database [30]. Clinical data and CT images were collected from VHS NSCLC patients under an Institutional Review Board (IRB)-approved protocol and merged with new NCSLC patient data from the TCIA database that includes lung CT images and clinical information of patients [31]. Since the TCIA is a publicly available database without patient identifiers, IRB approval is not required. Among the two TCIA datasets, R01 and AMC, the AMC dataset was excluded because it did not have pertinent clinical information such as the TN stage. All subjects from all datasets were pathologically confirmed to have lung squamous cell carcinoma (LUSC) or lung adenocarcinoma (LUAD). Patients with a history of multiple surgeries, with missing information related to the research, or referred to as “inadequate cases” by the experts were excluded. The demographic and clinical characteristics of all 326 NSCLC patients are provided in Table 1, including the age, TN stage, and recurrence.

### 2.2. Image Processing

**Figure 1:**
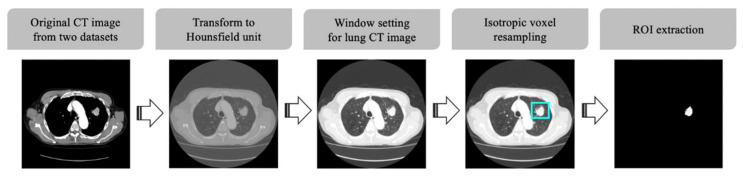
CT image preprocessing pipeline.

Lung CT images of the dataset utilized in this study were acquired with various multidetector CT (MDCT) scanners. To prevent problematic spatial information lacking uniformity, when using convolution layers for deep learning, CT scans were preprocessed as summarized in Figure 1.

First, CT scans were standardized to Hounsfield Units (HU), which is a quantitative indicator of the X-ray attenuation degree for each pixel in a CT image. The intensity in CT image is related to tissue density and can be measured in HU, allowing for the direct comparison of images from different sources [20]. After standardizing the pixel values of the target images into HU, a defined HU value was selected to display the images in grayscale. In our study, the window level and window width were set to −600 and 1500 HU, respectively, the values regularly used for general lung CT image display [32]. Furthermore, isotropic voxel resampling was performed to compensate for CT slide thickness variation and ensure uniform isotropic resolution of all CT images. Finally, regions of interest (ROI) images to be used for CNN analysis were generated based on the tumor segmentation labels, and all non-tumor pixels in the ROI images were set to zero.

### 2.3. Feature Extraction and Selection

Clinical and pathologic characteristics/features including clinical characteristics, laboratory results, and pathologic conditions were collected from the patient records. Of the features available for examination, only the clinical features routinely obtained from various institutions were considered. Additionally, we excluded features with a missing value in >25% of cases at either institution. Among the remaining pathologic features, we utilized some traditionally accepted variables with well-known prognostic power; these included histology, TN stage, age, and overall stage [33]. Specifically, visceral pleural invasion and lymphovascular invasion have been found to be two critical indicators for risk of recurrence [29,34]. The final clinical features utilized in our predictive model are listed in Table 2.

Next, to extract HCR features, volumetric tumor contours in lung CT images from VHS medical center and TCIA were manually segmented by two experienced radiologists (with more than 10 years of experience in lung diagnosis) of VHS medical center and Ewha Womans University Seoul Hospital, respectively, following the RTOG 1106 contouring guideline [35,36]. A total of 1668 HCR features were extracted from 3D ROI using Pyradiomics [37], which is an open-source package in Python. HCR features can be divided into four categories, shape, first-order statistics, second-order statistics, and high-order statistics, which are obtained after applying filters or mathematical transforms to the images. Attempting to use all the numerous extracted HCR features makes developing effective classification models very challenging. This is due to a high probability that many features are redundant and/or highly correlated, which can lead to over-fitting the results and affect the final performance of the network. Furthermore, Cox-proportional hazard regression was employed to identify the subset of HCR features with the most discriminating power for a given task and the greatest reduction in the data dimension. This analysis was done in a univariate way by applying a 0.05 *p*-value threshold. As such, we excluded variables with high correlation (>0.95) and near-zero variance, as they did not provide advantageous information to our model. As a result, 157 out of 1668 HCR features were selected as training variables. Table 3 shows the details of the selected HCR features by category.

Lastly, DLR features were not explicitly designed like HCR features. However, convolutional neural networks trained on CT images to perform a pre-defined task of predicting cancer recurrence were able to learn filters that function as edge detectors in the early layers. In the input, deeper layers respond to more complex patterns that resemble texture, shapes, or compositions of earlier features. The DLR features obtained from deep layers contain more abstract predictive patterns of CT images, compared with the clinical and HCR features. The extracted DLR features went through the rest of the deep networks to output cancer recurrence probability [0, 1].

### 2.4. Neural Network-Based Cancer Prediction Model

**Figure 2:**
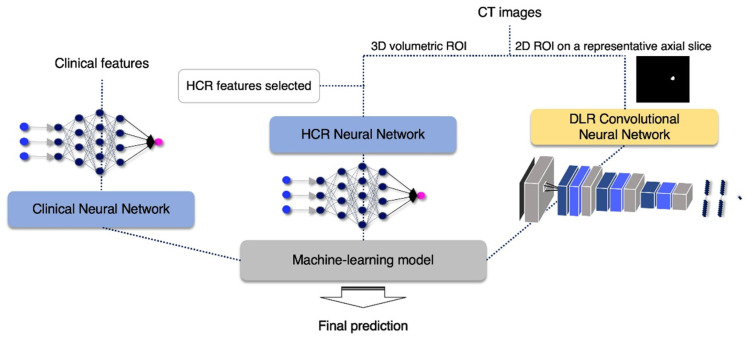
Flow chart of the proposed algorithm.

To obtain a well-organized ensemble model, we initially pretrained three neural network-based models with clinical data, HCR, and DLR, respectively, as shown in Figure 2. The clinical neural network was trained with the clinical features listed in Table 2, and the HCR neural network was learned from the selected HCR features shown in Table 3. The two neural network architectures mainly consisted of dense layers, batch normalization layers, dropout layers, and rectified linear units (ReLU), excluding the last layer, which underwent sigmoid optimization with Adam. While the clinical neural network had three pairs of dense-batch normalization-activation-dropout with the shape of increasing and decreasing the number of nodes, the HCR neural network had four pairs without batch normalization layer.

**Figure 3:**
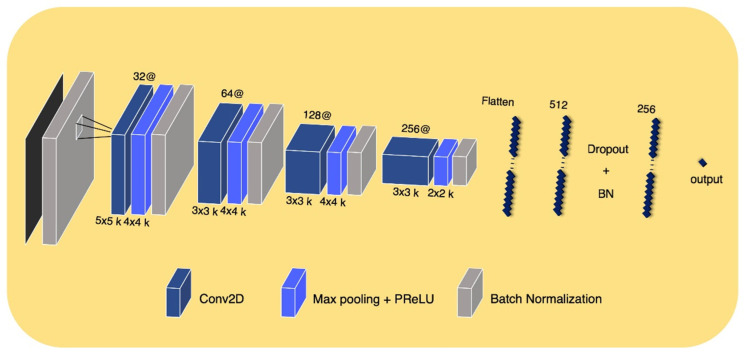
DLR convolutional neural network architectur.

The DLR convolutional neural network model input was a 2D ROI image on a representative axial slice, where tumor pixels were the largest within the entire volume of the CT image of each patient. Using this network, a large diversity of features was learned, especially through non-linearity operations, as images were passing through convolutional blocks. The DLR convolutional neural network contained four convolution layers and two fully connected layers. The Max-pooling layer, parametric PReLU as an activation function, and batch normalization layer followed after convolution layers, and 0.5 of dropout probability was included between the fully connected layers to avoid overfitting. A detailed composition is shown in Figure 3. The training was optimized with stochastic gradient descent and began with an initial learning rate of 0.01 and a momentum of 0.5. This learning rate fell 0.8×, to at most 0.0005 when the validation loss stopped diminution for a time. The model provided automatically augmented images from the original input images. These images were generated in simple ways including rotating (~20°), flipping (vertically or horizontally), or shifting.

In the end, a machine learning-based analyzer was employed to combine the predictive outputs (cancer recurrence probability [0, 1]) of each neural network-based model to improve the accuracy of prior models (i.e., ensemble learning). The combined outputs went through a different machine learning-based ensemble analyzer for making the final decision. The final analyzer was determined based on the performance of several representative analyzers. These tested analyzers included random forests, logistic regression, support vector machine, Gaussian naive Bayes, linear discriminant analysis, quadratic discriminant analysis, and hard and soft voting classifier. The research workflow is demonstrated in Figure 2.

### 2.5. Model Assessment

We applied a 5-fold cross-validation method with shuffled training data and evaluated the predictive abilities of each designed neural network with clinical features, HCR, and DLR, as well as the final proposed ensemble model. Moreover, we compared the performance of our proposed three neural network models with pre-existing models. The Cox proportional hazards (PH) model [38], traditionally used to predict the clinical outcomes or hazard functions corresponding to specific time units [39], was employed to compare predictive power using clinical variables. Selected HCR and DLR of CT images were utilized by more recent machine learning-based prediction models, random survival forest [40] and convolutional neural network [41], respectively. In addition, a neural network model with TN staging was developed to use as a baseline for performance evaluation.

The performance of the baseline model (TN staging), individual data feature models (clinical data, HCR, and DLR), and the all three data feature model (clinical data, HCR, and DLR) were evaluated using quantitative metrics including F1 score, precision, recall, and accuracy from the confusion matrix. Precision was defined as the ratio of the actual positive of the model determined to be positive. Recall was determined as the ratio of how many positive observations were found in the actual positive observations. Thus, in general, because the precision and recall values of the algorithms are inversely related, they should be considered simultaneously for evaluating the classification performance. Moreover, the F1 score is the weighted average of precision and recall, thereby making it the single numeric representation of the algorithm’s precision and recall. Finally, accuracy refers to the ability of the algorithm to precisely judge positive and negative.

The results of the 5-fold cross-validation were also used to draw an ROC curve with an AUC value for each model under the same condition. An ROC curve is a plot of the true positive rate against the false-positive rate at various threshold settings. The ROC curve is crucial for future medical applications because it evaluates the diagnostic ability of tests to discriminate a subject’s true state and finds optimal cut off values [42,43,44,45].

To determine if there was a significant difference between the patients classified by the model, we generated Kaplan–Meier curves with a log-rank test to analyze using one of the 5-fold results. The Kaplan–Meier estimates are one of the best methods used to calculate the proportion of patients who do not relapse or survive a certain period of time after treatment [46]. Lower *p*-values of log-rank test are correlated with less Kaplan–Meier curve overlaps, and clearer distinction.

## 3. Results

### 3.1. Quantitative Measurements

The 5-fold cross-validation quantitative results including F1-score, precision, recall, and accuracy are displayed in Table 4. Our results show that our proposed ensemble model that incorporated clinical, HCR, and DLR performed better than all other current methods. Notably, the conventional method, the Cox PH model, underperformed the most extensively. Interestingly, clinical and HCR features had better prognostic power of recurrence than DLR features. Of the machine learning algorithms tested, linear discriminant analysis (LDA), quadratic discriminant analysis (QDA), and logistic regression (LR) were most successful in predicting recurrence.

### 3.2. ROC Curve with AUC Value

**Figure 4:**
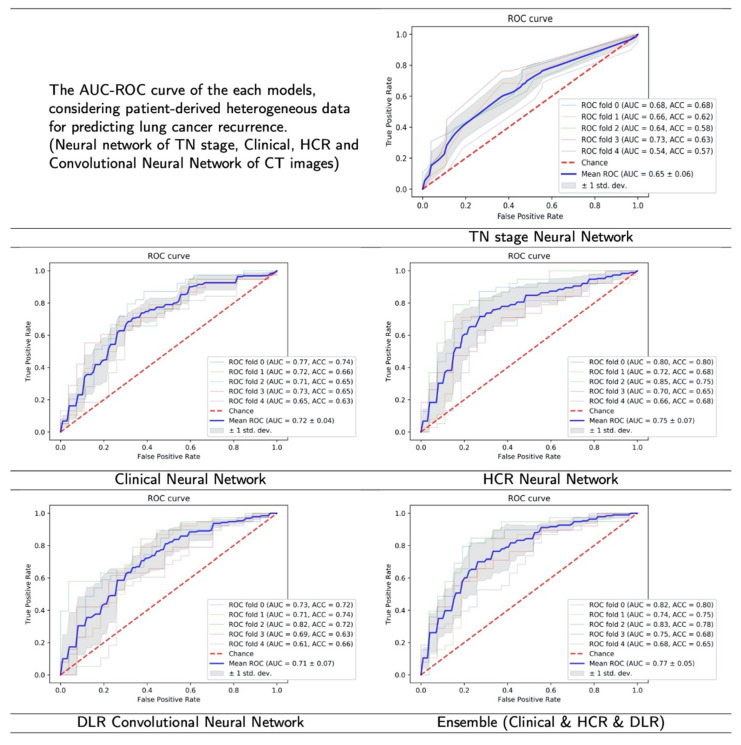
The result figures of ROC curve with AUC value. The highest average AUC value was recorded in the proposed model (ensemble), followed by HCR, Clinical, DLR and TN stage.

Based on the ROC curve with AUC value, experimental results with the 5-fold cross-validation indicate that the proposed ensemble algorithm had the highest predictive power when the clinical data, HCR, and DLR were included together, as opposed to the use of individual features (Figure 4). The proposed HCR neural network model showed the next best performance with a slightly lower value of AUC, compared to the ensemble model.

### 3.3. Kaplan–Meier Curve

**Figure 5:**
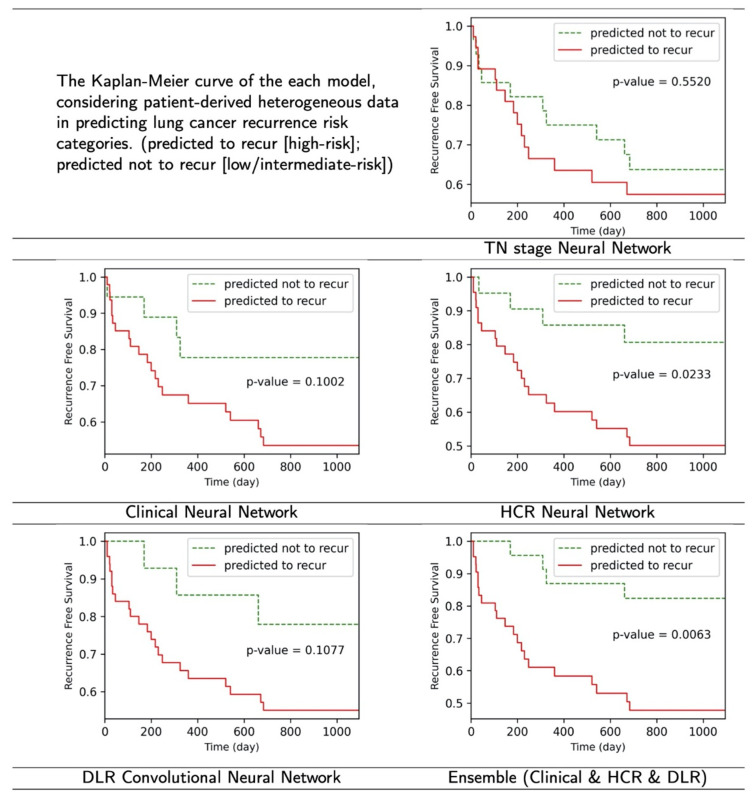
The result figures of Kaplan–Meier curve. The proposed ensembled model and HCR neural network model showed meaningful results (*p* < 0.05), while others did not in the log-rank test.

Figure 5 shows the Kaplan–Meier curves derived from each model’s lung cancer recurrence risk. The predicted to recur group indicates a high risk of recurrence, whereas the predicted not to recur group reveals a low or intermediate-risk. Of the 65–66 test data in one-fold, an average of 9.8 cases (SD 1.7, 15.1% of one test set) of the incorrect predictions from the TN stage neural network (baseline), 7.0 cases (SD 0.9, 10.8% of one test set) of the incorrect predictions from the Clinical neural network, 3.2 cases (SD 1.9, 4.9% of one test set) of the incorrect predictions from the HCR neural network, and 6.6 cases (SD 2.41, 10.2% of done test set) of the incorrect predictions from the DLR convolutional neural network were correctly classified by our proposed ensemble model.

## 4. Discussion

Despite surgical resection being the major treatment for NSCLC, adjuvant chemotherapy is regularly employed as standard post-surgical care. Adjuvant chemotherapy is critical due to the high probability of occult micro-metastases present at the time of surgical intervention [47]. Importantly, post-surgery chemotherapy attempts to eliminate any metastasized cancer cells and prevent recurrence. Although chemotherapy is correlated with an increase in overall survival in early-stage NSCLC patients [48,49,50], questions about whether adjuvant chemotherapy should be accompanied in cases of patients with high predictive risk of recurrence still remains.

Considering that the recurrence of NSCLC typically occurs at sites distal to the original tumor [51,52], patients at high risk of recurrence should be treated similarly to advanced NSCLC patients. As such, the ability to predict recurrence is very critical in early-stage (i.e., IA) patients. In these cases, patients with high probabilities of recurrence can be treated with adjuvant therapies as prevention measures. Conversely, with high powered predictive models, patients with a low predictive risk of recurrence can avoid unnecessary adjuvant therapies. Therefore, it is critical to have powerful predictive models to differentiating high risk and low-risk patients. With this information, patients can make an informed decision about their treatment options. The ensemble model we proposed here showed an 11.69% higher accuracy than the TN staging-based algorithm (baseline) as shown in Table 4. Additionally, the proposed model was able to accurately predict 15.1% of cases where the baseline algorithm failed (see Section 3.3). The high accuracy of our algorithm in predicting recurrence can aid clinicians in guiding appropriate treatment strategies for each patient.

Considering that the TN staging has limitations such as patient to patient recurrence rate variations [29], this study attempted to develop and validate an NSCLC recurrence prediction model for patients with surgically resected lungs. Using the same data sets, the three proposed neural network-based models (Clinical neural network, HCR neural network, and DLR convolutional neural network) were more accurate in predicting recurrence than existing representative models [38,40,41].

Moreover, we hypothesized that deep learning models learned using valid clinical variables, features extracted explicitly (i.e., HCR) and automatically induced (i.e., DLR) from medical images, could be applied back to the ensemble model to produce more accurate and refined results. Our results demonstrate that the proposed ensemble model that utilized all data (clinical data, HCR, and DLR) outperformed proposed models that used individual data. For the case of this study, the proposed final ensemble-based model was able to accurately predict the recurrence for the cases of 4.9% to 15.1%, where the models trained with single data-type features failed to predict. Therefore, the proposed method can help more accurately identify patients with a high risk of recurrence, and thus can significantly reduce erroneous postoperative cancer treatment decisions due to incorrect recurrence prediction. According to Table 4, the computational complexity of DLR networks is much higher than the other single-modality and TN stage (baseline) models. HCR networks utilize more features and therefore have slightly higher computational complexity than TN (baseline) and clinical models. In addition, the proposed algorithm integrates multimodal features through a machine learning-based analyzer, thus exhibiting computational complexity similar to that of DLR model. Although our proposed ensemble models have higher model complexity compared to the baseline, the inference time per image with increasing complexity is several milliseconds. Given that it can take up to minutes for a clinician to review a patient’s multimodal information and draw a comprehensive conclusion, the increase of several milliseconds is considered negligible.

Proper cancer prognosis and treatment is a very difficult and complex task for oncologists. Oncologists must choose from a wide range of treatment options to fit a patient’s clinical and pathological data [53]. Therefore, the algorithm for predicting relapse using only TN stage information or CT images is far removed from actual clinical decision making; however, the proposed ensemble model that utilizes all three available features (clinical data, HCR, and DLR) is more clinically relevant.

Several limitations of our study need to be addressed. First, we did not account for the possibility of overlapping among heterogeneous features. While we removed features with a high correlation within the HCR features, the correlation between each network input feature was not analyzed. This is likely to impede the final model performance and involves a process that is less efficient. Second, the DLR convolutional neural network model used a representative 2D slice rather than a full 3D volumetric image, indicating a possible loss of information. However, the effective through-plane (z)-resolution is typically much lower than the in-plane resolution of the axial slice, thus, the use of 2D slices may still produce better results [41]. Several studies have attempted to improve the *z*-axis resolution [54,55,56]; therefore, as research in this field develops, the algorithm can be improved upon by incorporating a 3D CT image. Thus, in future studies, we plan to develop a more robust model that utilizes a complete 3D CT image, as well as eliminating redundancy between heterogeneous data. Furthermore, we plan to develop a model that utilizes all three different neural networks simultaneously.

## 5. Conclusions

Here, we present a machine learning-based ensemble methodology with various NSCLC patients’ clinical and pathological data that predicts cancer recurrence after surgical resection. Specifically, we utilized clinical variables and radiomic features, which included both human-designed (HCR) and automatically learned and extracted by the DLR convolutional neural networks features. Each of these showed meaningfully significant predictive power. The three proposed models for clinical data, HCR, and DLR more accurately predicted recurrence than existing representative models using the same individual data. Each neural network-based model produced outcomes that were combined and subsequently put through a different machine learning-based ensemble analyzer to further improve accuracy and making the final decision. The overall performance of our suggested ensemble model was greater than models that did not use all three data types (clinical data, HCR, and DLR). Moreover, the proposed model was both efficient and robust, as evidenced by the k-fold cross-validation. We believe the better prediction capabilities of our system can improve the care of NSCLC patients and allow for more efficient decision making for treatment.

## Figures and Tables

**Table 1:** Demographic characteristics.

Characteristics	Number of Patients (%)	
Institution		
VHS Medical Center	224	(68.7%)
TCIA (R01)	102	(31.3%)
Age (70.92 ± 7.8)		
Age < 60	17	(5.2%)
Age ≥ 60	309	(94.8%)
Histology		
Adenocarcinoma	167	(51.2%)
Squamous cell carcinoma	159	(48.8%)
T stage		
Tis, T1	146	(44.8%)
T2	149	(45.7%)
T3, T4	31	(9.5%)
N stage		
N0	210	(64.4%)
N1	62	(19.0%)
N2	54	(16.6%)
Recurrence		
Recurred	193	(59.2%)
Not recurred	133	(40.8%)

**Table 2:** Clinical variable used in the first neural network model.

Clinical Features
LUAD/LUSC, Age, Overall stage, T stage (T1, T2, T3), N stage (N0, N1, N2),
Pathology-visceral pleural (+/−), Pathology-lymphovascular invasion (+/−)

**Table 3:** Handcrafted radiomic features used in the second neural network model.

Handcrafted Radiomic Features by Category	
First Order	
Shape	5
Second order (texture features)	
gray level co-occurrence (GLCM)	4
gray level run-length (GLRLM)	3
gray level size zone (GLSZM)	2
gray level dependence matrix (GLDM)	2
neighborhood gray tone difference matrix (NGTDM)	1
High order	140

**Table 4:** Mean and standard deviation values of the 5-fold cross validation in terms of F1 score, precision, recall and accuracy. The first three rows reported the implementation results of other competitive methods using each of our dataset. Type of machine learning model was recorded if the model of the row was ensembled. The number of trainable parameters is displayed in thousands in the rightmost column. The models that shows significant difference with the TN stage (baseline) for all evaluation metrics are marked with an asterisk (*).

	F1 Score	Precision	Recall	Accuracy	Model	Trainable Parameters
Clinical (David Cox) [38]	68.11 (±2.4)	56.51 (±1.4)	85.84 (±5.5)	52.90 (±2.5)	Cox PH	-
HCR (Wen Yu et al.) [40]	72.19 (±3.4)	63.78 (±2.1)	83.23 (±5.5)	62.44 (±3.8)	RSF	-
DLR (André Diamant et al.) [41]	74.55 (±5.4)	69.12 (±6.3)	81.12 (±5.2)	67.35 (±7.1)	CNN	916 K
TN stage (baseline)	65.67 (±5.1)	70.09 (±8.0)	63.81 (±10.6)	61.54 (±3.8)	NN	1 K
Clinical	73.54 (±2.4)	68.99 (±5.1)	79.07 (±2.2)	66.46 (±3.8)	NN1	1 K
HCR	76.61 (±4.7)	73.07 (±5.2)	80.61 (±4.7)	71.08 (±5.7)	NN2	10 K
DLR	76.28 (±4.9)	70.11 (±3.1)	84.80 (±11.2)	69.54 (±4.2)	CNN	1046 K
Clinical & HCR *	77.58 (±5.0)	75.29 (±4.1)	80.08 (±6.4)	72.92 (±5.6)	QDA	11 K
Clinical & DLR *	76.86 (±4.7)	71.03 (±3.7)	83.75 (±6.1)	70.46 (±5.6)	LR	1047 K
HCR & DLR *	77.65 (±5.0)	73.70 (±4.5)	82.19 (±6.6)	72.31 (±5.9)	LR	1056 K
Clinical & HCR & DLR *	77.79 (±5.3)	75.71 (±4.8)	80.08 (±6.4)	73.23 (±6.0)	LDA	1057 K

## Data Availability

Not applicable.

## Abbreviations

The following abbreviations are used in this manuscript:

NSCLC	non-small cell lung cancer
LUAD	lung adenocarcinoma
LUSC	lung squamous cell carcinoma
TN	tumor node
HCR	handcrafted radiomics
DLR	deep learning-based radiomics
CNN	convolutional neural network
PH	proportional hazards
SD	standard deviation

## References

[1] Parmar C., Leijenaar R.T., Grossmann P., Rios Velazquez E., Bussink J., Rietveld D., Rietbergen M.M., Haibe-Kains B., Lambin P., Aerts H.J. (2015). Radiomic feature clusters and prognostic signatures specific for lung and head & neck cancer. Sci. Rep..

[2] Jemal A., Bray F., Center M.M., Ferlay J., Ward E., Forman D. (2011). Global cancer statistics. CA Cancer J. Clin..

[3] Chen Y.-Y., Huang T.-W., Tsai W.-C., Lin L.-F., Cheng J.-B., Chang H., Lee S.-C. (2014). Risk factors of postoperative recurrences in patients with clinical stage I NSCLC. World J. Surg. Oncol..

[4] Ponn R.B., Lo Cicero J., Daly B.D. (2005). Surgical treatment of non-small cell lung cancer. Gen. Thorac. Surg..

[5] Wright G., Manser R.L., Byrnes G., Hart D., Campbell D.A. (2006). Surgery for non-small cell lung cancer: Systematic review and meta-analysis of randomised controlled trials. Thorax.

[6] Strauss G.M. (2005). Adjuvant chemotherapy of lung cancer: Methodologic issues and therapeutic advances. Hematol./Oncol. Clin..

[7] Herbst R., Heymach J. (2008). liPPman Sm. Lung Cancer. N. Engl. J. Med..

[8] Song J., Liu Z., Zhong W., Huang Y., Ma Z., Dong D., Liang C., Tian J. (2016). Non-small cell lung cancer: Quantitative phenotypic analysis of CT images as a potential marker of prognosis. Sci. Rep..

[9] Papadimitrakopoulou V. (2019). PC03.03 Future Strategies in Early Stage EGFR-mut NSCLC. J. Thorac. Oncol..

[10] Scagliotti G.V., Fossati R., Torri V., Crino L., Giaccone G., Silvano G., Martelli M., Clerici M., Cognetti F., Tonato M. (2003). Randomized study of adjuvant chemotherapy for completely resected stage I, II, or IIIA non–small-cell lung cancer. J. Natl. Cancer Inst..

[11] Laskin J.J. (2005). Adjuvant chemotherapy for non-small cell lung cancer: The new standard of care. Future Med..

[12] Elston C.W., Ellis I.O. (1991). Pathological prognostic factors in breast cancer. I. The value of histological grade in breast cancer: Experience from a large study with long-term follow-up. Histopathology.

[13] Harpole D.H., Herndon J.E., Wolfe W.G., Iglehart J.D., Marks J.R. (1995). A prognostic model of recurrence and death in stage I non-small cell lung cancer utilizing presentation, histopathology, and oncoprotein expression. Cancer Res..

[14] Harpole D.H., Herndon J.E., Young W.G., Wolfe W.G., Sabiston D.C. (1995). Stage I nonsmall cell lung cancer. A multivariate analysis of treatment methods and patterns of recurrence. Cancer.

[15] Vansteenkiste J., Crino L., Dooms C., Douillard J.-Y., Faivre-Finn C., Lim E., Rocco G., Senan S., Van Schil P., Veronesi G. (2014). 2nd ESMO Consensus Conference on Lung Cancer: Early-stage non-small-cell lung cancer consensus on diagnosis, treatment and follow-up. Ann. Oncol..

[16] Crino L., Weder W., Van Meerbeeck J., Felip E. (2010). Early stage and locally advanced (non-metastatic) non-small-cell lung cancer: ESMO Clinical Practice Guidelines for diagnosis, treatment and follow-up. Ann. Oncol..

[17] Detterbeck F.C., Boffa D.J., Tanoue L.T. (2009). The new lung cancer staging system. Chest.

[18] van Riel S.J., Sánchez C.I., Bankier A.A., Naidich D.P., Verschakelen J., Scholten E.T., de Jong P.A., Jacobs C., van Rikxoort E., Peters-Bax L. (2015). Observer variability for classification of pulmonary nodules on low-dose CT images and its effect on nodule management. Radiology.

[19] Arena E.A., Bilchik A.J. (2013). What is the optimal means of staging colon cancer?. Adv. Surg..

[20] Bizzego A., Bussola N., Salvalai D., Chierici M., Maggio V., Jurman G., Furlanello C. Integrating deep and radiomics features in cancer bioimaging. Proceedings of the 2019 IEEE Conference on Computational Intelligence in Bioinformatics and Computational Biology (CIBCB).

[21] Aerts H.J., Velazquez E.R., Leijenaar R.T., Parmar C., Grossmann P., Carvalho S., Bussink J., Monshouwer R., Haibe-Kains B., Rietveld D. (2014). Decoding tumour phenotype by noninvasive imaging using a quantitative radiomics approach. Nat. Commun..

[22] Cetin K., Ettinger D.S., Hei Y.-J., O’Malley C.D. (2011). Survival by histologic subtype in stage IV nonsmall cell lung cancer based on data from the Surveillance, Epidemiology and End Results Program. Clin. Epidemiol..

[23] Coroller T.P., Grossmann P., Hou Y., Velazquez E.R., Leijenaar R.T., Hermann G., Lambin P., Haibe-Kains B., Mak R.H., Aerts H.J. (2015). CT-based radiomic signature predicts distant metastasis in lung adenocarcinoma. Radiother. Oncol..

[24] Lambin P., Rios-Velazquez E., Leijenaar R., Carvalho S., Van Stiphout R.G., Granton P., Zegers C.M., Gillies R., Boellard R., Dekker A. (2012). Radiomics: Extracting more information from medical images using advanced feature analysis. Eur. J. Cancer.

[25] Orlhac F., Nioche C., Soussan M., Buvat I. (2017). Understanding changes in tumor texture indices in PET: A comparison between visual assessment and index values in simulated and patient data. J. Nucl. Med..

[26] Chalkidou A., O’Doherty M.J., Marsden P.K. (2015). False discovery rates in PET and CT studies with texture features: A systematic review. PLoS ONE.

[27] Kato S., Nambu A., Onishi H., Saito A., Kuriyama K., Komiyama T., Marino K., Araki T. (2010). Computed tomography appearances of local recurrence after stereotactic body radiation therapy for stage I non-small-cell lung carcinoma. Jpn. J. Radiol..

[28] Huang K., Dahele M., Senan S., Guckenberger M., Rodrigues G.B., Ward A., Boldt R.G., Palma D.A. (2012). Radiographic changes after lung stereotactic ablative radiotherapy (SABR)–can we distinguish recurrence from fibrosis? A systematic review of the literature. Radiother. Oncol..

[29] Uramoto H., Tanaka F. (2014). Recurrence after surgery in patients with NSCLC. Transl. Lung Cancer Res..

[30] Clark K., Vendt B., Smith K., Freymann J., Kirby J., Koppel P., Moore S., Phillips S., Maffitt D., Pringle M. (2013). The Cancer Imaging Archive (TCIA): Maintaining and operating a public information repository. J. Digit. Imaging.

[31] Bakr S., Gevaert O., Echegaray S., Ayers K., Zhou M., Shafiq M., Zheng H., Benson J.A., Zhang W., Leung A.N. (2018). A radiogenomic dataset of non-small cell lung cancer. Sci. Data.

[32] Romans L. (2018). Computed Tomography for Technologists: A comprehensive Text.

[33] Crvenkova S. (2015). Prognostic factors and survival in non-small cell lung cancer patients treated with chemoradiotherapy. Open Access Maced. J. Med. Sci..

[34] Shiono S., Abiko M., Sato T. (2011). Positron emission tomography/computed tomography and lymphovascular invasion predict recurrence in stage I lung cancers. J. Thorac. Oncol..

[35] Kong F., Machtay M., Bradley J., Ten Haken R., Xiao Y., Matuszak M., Hirsh V., Pryma D. (2012). RTOG 1106/ACRIN 6697: Randomized Phase II Trial of Individualized Adaptive Radiotherapy Using during Treatment FDG-PET/CT and Modern Technology in Locally Advanced Non-Small Lung Cancer (NSCLC).

[36] Kong F.-M., Ten Haken R.K., Schipper M., Frey K.A., Hayman J., Gross M., Ramnath N., Hassan K.A., Matuszak M., Ritter T. (2017). Effect of midtreatment PET/CT-adapted radiation therapy with concurrent chemotherapy in patients with locally advanced non–small-cell lung cancer: A phase 2 clinical trial. JAMA Oncol..

[37] Van Griethuysen J.J., Fedorov A., Parmar C., Hosny A., Aucoin N., Narayan V., Beets-Tan R.G., Fillion-Robin J.-C., Pieper S., Aerts H.J. (2017). Computational radiomics system to decode the radiographic phenotype. Cancer Res..

[38] Cox D.R. (1972). Regression models and life-tables. J. R. Stat. Soc. Ser. B.

[39] Lee B., Chun S.H., Hong J.H., Woo I.S., Kim S., Jeong J.W., Kim J.J., Lee H.W., Na S.J., Beck K.S. (2020). DeepBTS: Prediction of recurrence-free survival of non-small cell lung cancer using a time-binned deep neural network. Sci. Rep..

[40] Yu W., Tang C., Hobbs B.P., Li X., Koay E.J., Wistuba I.I., Sepesi B., Behrens C., Canales J.R., Cuentas E.R.P. (2018). Development and validation of a predictive radiomics model for clinical outcomes in stage I non-small cell lung cancer. Int. J. Radiat. Oncol. Biol. Phys..

[41] Diamant A., Chatterjee A., Vallières M., Shenouda G., Seuntjens J. (2019). Deep learning in head & neck cancer outcome prediction. Sci. Rep..

[42] Hanley J.A., McNeil B.J. (1982). The meaning and use of the area under a receiver operating characteristic (ROC) curve. Radiology.

[43] Hanley J.A., McNeil B.J. (1983). A method of comparing the areas under receiver operating characteristic curves derived from the same cases. Radiology.

[44] Kumar R., Indrayan A. (2011). Receiver operating characteristic (ROC) curve for medical researchers. Indian Pediatr..

[45] Hajian-Tilaki K. (2013). Receiver operating characteristic (ROC) curve analysis for medical diagnostic test evaluation. Casp. J. Intern. Med..

[46] Goel M.K., Khanna P., Kishore J. (2010). Understanding survival analysis: Kaplan-Meier estimate. Int. J. Ayurveda Res..

[47] De Ruysscher D., Faivre-Finn C., Nackaerts K., Jordan K., Arends J., Douillard J.-Y., Ricardi U., Peters S. (2020). Recommendation for supportive care in patients receiving concurrent chemotherapy and radiotherapy for lung cancer. Ann. Oncol..

[48] Wada H., Hitomi S., Teramatsu T. (1996). Adjuvant chemotherapy after complete resection in non-small-cell lung cancer. West Japan Study Group for Lung Cancer Surgery. J. Clin. Oncol..

[49] Guerra J.L., Gomez D., Lin S., Levy L., Zhuang Y., Komaki R., Jaen J., Vaporciyan A., Swisher S., Cox J. (2013). Risk factors for local and regional recurrence in patients with resected N0–N1 non-small-cell lung cancer, with implications for patient selection for adjuvant radiation therapy. Ann. Oncol..

[50] Burdett S., Pignon J.P., Tierney J., Tribodet H., Stewart L., Le Pechoux C., Aupérin A., Le Chevalier T., Stephens R.J., Arriagada R. (2015). Adjuvant chemotherapy for resected early-stage non-small cell lung cancer. Cochrane Database Syst. Rev..

[51] Yamashita T., Uramoto H., Onitsuka T., Ono K., Baba T., So T., So T., Takenoyama M., Hanagiri T., Oyama T. (2010). Association between lymphangiogenesis-/micrometastasis-and adhesion-related molecules in resected stage I NSCLC. Lung Cancer.

[52] Nakagawa M., Uramoto H., Oka S., Chikaishi Y., Iwanami T., Shimokawa H., So T., Hanagiri T., Tanaka F. (2012). Clinical significance of IGF1R expression in non–small-cell lung cancer. Clin. Lung Cancer.

[53] Glatzer M., Panje C.M., Sirén C., Cihoric N., Putora P.M. (2020). Decision making criteria in oncology. Oncology.

[54] Tsukagoshi S., Ota T., Fujii M., Kazama M., Okumura M., Johkoh T. (2007). Improvement of spatial resolution in the longitudinal direction for isotropic imaging in helical CT. Phys. Med. Biol..

[55] McCollough C.H., Leng S., Sunnegardh J., Vrieze T.J., Yu L., Lane J., Raupach R., Stierstorfer K., Flohr T. (2013). Spatial resolution improvement and dose reduction potential for inner ear CT imaging using az-axis deconvolution technique. Med. Phys..

[56] Higaki T., Nakamura Y., Tatsugami F., Nakaura T., Awai K. (2019). Improvement of image quality at CT and MRI using deep learning. Jpn. J. Radiol..

